# Development of a framework of potential adverse effects of interventions to improve critical thinking about health choices: A mixed methods study.

**DOI:** 10.12688/f1000research.158042.1

**Published:** 2024-10-31

**Authors:** Matt Oxman, Faith Chelagat Chesire, Michael Mugisha, Ronald Ssenyonga, Benson Ngatia, Allen Nsangi, Simon Lewin, Jenny Moberg, Nelson Sewankambo, Margaret Kaseje, Monica Melby-Lervåg, Atle Fretheim, Andrew David Oxman, Sarah Rosenbaum

**Affiliations:** 1 Centre for Epidemic Interventions Research, Norwegian Institute of Public Health, Oslo, Norway; 2 Faculty of Health Sciences, Oslo Metropolitan University, Oslo, Norway; 3 Tropical Institute of Community Health and Development, Kisumu, Kisumu County, Kenya; 4 Institute of Health and Society, University of Oslo, Oslo, Norway; 5 School of Public Health, University of Rwanda College of Medicine and Health Sciences, Kigali, Kigali City, Rwanda; 6 Makerere University College of Health Sciences, Kampala, Central Region, Uganda; 7 Faculty of Medicine and Health Sciences, Norwegian University of Science and Technology, Ålesund, Norway; 8 Health Systems Research Unit, South African Medical Research Council, Cape Town, South Africa; 9 Centre for Research on Special Needs Education and Inclusion,, University of Oslo, Oslo, Norway

**Keywords:** Adverse effects, Harms; Side effects, Unintended effects, Unanticipated effects, Unexpected effects, Public health, Critical health literacy

## Abstract

**Background:**

People need critical thinking skills to make well-informed health choices and avoid waste and unnecessary suffering. However, educational interventions can have adverse effects, which is often overlooked. We created a framework of potential harms of interventions to improve critical thinking about health choices. The objective was to identify potential effects and articulate potential mechanisms. We intended to use the framework to inform the development and evaluation of an intervention in Kenya, Rwanda, and Uganda.

**Methods:**

We created an initial framework drawing on two earlier frameworks. We surveyed external experts using Likert-scale and open-ended items, including researchers, educators, and others, in a variety of relevant fields. We emailed the survey to an international advisory network, and all other experts recommended by respondents. We analyzed the quantitative data using descriptive statistics. We conducted a thematic analysis of the qualitative data. We revised the framework based on those results. To identify any effects missing from the revised framework, we individually interviewed a convenience sample of one teacher from each of Kenya, Rwanda, and Uganda.

**Results:**

We received responses from 38 of 70 external experts (54%). The quantitative survey data suggested respondents agreed with the initial framework overall. However, the qualitative data led to substantial revisions. The revised framework consists of categories of adverse outcomes; outcomes within each category; sub-outcomes; individuals, groups, and populations that might experience each adverse outcome; beneficial outcomes corresponding with adverse outcomes; and potential mechanisms of adverse effects. There are six categories: decision-making harms, psychological harms, equity harms, group and social harms, waste, and other harms. In the interviews with teachers, we did not identify any effects missing from the revised framework.

**Conclusions:**

As far as we know, the framework is the first tool of its kind for education research. It can be improved upon and adapted.

## Introduction

### Critical thinking about health choices

The Covid-19 pandemic exacerbated an overabundance of health information, including both reliable and unreliable claims and research evidence about the effects of health interventions. ^1, 2^ A health intervention is any action intended to improve or maintain the health of an individual, group, or population, including “modern”, “academic”, “conventional”, or “Western” medicine; “alternative”, “complementary”, “traditional”, or “natural” medicine; screening; surgery; devices and other equipment; non-medical first aid; physical therapy; diet, exercise, and lifestyle interventions; psychotherapy; and health systems and policies.

People need basic critical thinking skills to assess the reliability of claims and evidence about the effects of health interventions and make well-informed health choices. ^3, 4^ However, many people, including clinicians and other health professionals, lack such skills. ^5– 8^ This is a barrier to evidence-based practice ^9, 10^ and might help explain overuse of harmful medical services ^11– 13^ and underuse of effective and safe services. ^14^ The problem is most pressing in low-income countries, where there are fewer resources to waste.

Teaching young people how to think critically about health choices is advantageous. They have more time to learn and less to unlearn compared to clinicians and other adults. Moreover, young people include coming generations of health professionals. Teachers and others have expressed a need for interventions to help secondary school students learn how to think critically about health choices. ^15– 20^

### Adverse effects in education and public health research

Harms of medical interventions is an established field of study, ^21, 22^ granted such harms are inadequately reported in the scientific literature, ^23^ and mass media. ^1^ It is arguably less recognized that interventions in other fields, including education, can also have adverse effects. ^24– 28^ Herein, we use “harm” and “adverse effect” synonymously to describe an increase in undesirable outcomes or decrease in desirable outcomes. Elsewhere, “harm” might refer only to physical injury, or be associated only with medicine. Therefore, we generally use “adverse effect”.

Evaluations of the intended effects of educational interventions can reveal a “paradoxical” effect: a decrease in outcomes that the intervention was intended to increase, or an increase in outcomes that the intervention was intended to decrease. ^29, 30^ For example, a trial of the school improvement program Achievement for All, in the United Kingdom, showed a lack of effect on some outcomes, such as attendance, as well as paradoxical effects on academic outcomes, such as reading progress. ^31^

Educational interventions can also have adverse effects that are not paradoxical. Zhao presents cases of potential “side effects” of educational interventions: adverse effects of interventions that also have intended effects. ^26, 27^ For example, critics of “direct instruction” argue that although this pedagogical approach might improve academic outcomes as intended, it might also have an adverse effect on creativity. ^26, 27^

In a systematic review, MO *et al*. assessed the extent to which potential adverse effects are included in studies of interventions intended to improve laypeople’s critical thinking about health choices. The report of the review is in production. The results suggest researchers often assume such interventions do not have adverse effects. Of the 29 included reports of quantitative studies, ^32– 60^ one included the assessment of a potential adverse effect. ^48^

To help think about potential harms of public health interventions, Lorenc and Oliver created a generic framework with five overarching categories: direct harms, psychological harms, equity harms, group and social harms, and opportunity harms. ^61^ More recently, Stratil *et al*. developed a framework to support researchers and decision-makers in anticipating and assessing adverse effects of public health interventions: the Consequences of Public Health Interventions (CONSEQUENT) framework. ^62^ It includes eight domains: health, health system, human rights, acceptability and adherence, equality, and equity, social and institutional, economic and resources, and the environment. Allen-Platt *et al*. developed a framework to help prevent and evaluate “failures” of educational interventions, which includes interventions being ineffective or having paradoxical effects, but not other adverse effects. ^63^

In a chapter of *Evidence Matters: Randomized Trials in Education Research*, Weiss suggests intervention designers should articulate the potential mechanisms of both beneficial and adverse effects, to facilitate evaluating early stages of the intervention, and to help focus the evaluation on the most important outcomes. ^28^ Similarly, Bonell *et al.* suggest public health researchers should develop logic models with potential mechanisms of adverse effects—“dark” logic models—given that public health interventions are evaluated and implemented in complex social systems, involving human agency, and the mechanisms might not be obvious. ^30^ To theorize potential adverse effects, they suggest three strategies: reflecting on potential unintended interactions between the agency of providers, recipients, or other stakeholders, *versus* social structures that enable or constrain that agency; to consider evidence about the effects of similar interventions; and to consult with stakeholders. ^30^

### Informed Health Choices project

As part of the Informed Health Choices (IHC) project, we have developed and evaluated an intervention to help secondary school students learn how to think critically about health information and choices. We have built on findings and experiences from developing and evaluating an intervention targeted at primary school children. ^53, 64, 65^ In Box 1, we describe the aim, development, and evaluation of the IHC secondary school intervention in more detail.

Box 1. **Aim, development, and evaluation of the Informed Health Choices (IHC) secondary school intervention.**

The IHC secondary school intervention is intended to improve students’ ability to understand and apply a prioritized set of concepts. ^66, 70^ The concepts come from the Key Concepts for Informed Health Choices framework, or IHC Key Concepts framework. ^3, 66, 70, 102^ The ability to apply the IHC Key Concepts is part of “health literacy”, more specifically “critical health literacy”. ^103^ We developed the intervention in Kenya, Rwanda, and Uganda. ^66^ To inform the development, we first studied the contexts for teaching critical thinking about health using digital resources, in those countries. ^18– 20^ The final intervention had two components: training teachers and providing them with digital resources. ^66^ The semester following the training, teachers were intended to deliver 10 lessons to students. The lessons were introduced in the training and outlined in the resources. Each lesson plan focuses on one or more of the prioritized concepts. We evaluated the intervention in cluster-randomized trials in Kenya, Rwanda, and Uganda, ^104– 106^ and process evaluations associated with the trials. ^78, 98– 100^

In the trial of the IHC primary school intervention we asked the teachers to record and report adverse outcomes they experienced or observed. ^52, 53^ None were reported. In the process evaluation associated with that trial, we did not observe any adverse outcomes, but in interviews, some teachers said they felt stress due to the added workload, and to teaching something new. ^64^ Teachers and parents expressed concern that the intervention might cause conflict between the children and adults, by causing children to challenge adults’ authority. ^64^ They said there were cases of children challenging adults’ authority, but not of conflicts. ^64^

Like with the primary school intervention, we have employed an iterative, human-centered design approach to develop the secondary school intervention, involving cycles of prototyping, user-testing, and piloting. ^66^ This approach is helpful for identifying problems that could potentially lead to adverse effects and addressing them during the development stage. ^65, 66^ Moreover, findings about potential adverse effects from the development and evaluation of the primary school intervention have informed the development of the secondary school intervention.

Given the positive findings from the evaluation of the primary school intervention, as well as our rigorous approach to developing the secondary school intervention—including use of what we have learned from the development and evaluation of the primary school intervention—we did not anticipate serious adverse effects of the secondary school intervention. However, there were limitations to the evaluation of potential adverse effects of the primary school intervention. For example, we did not iteratively develop the framework used in that evaluation. ^64, 67^ Moreover, the secondary school intervention might have adverse effects that we failed to consider in the design process, for example because of social desirability bias, ^68^ or because of differences between schools that participated in piloting *versus* schools in the trials. The bottom line is that adverse effects are always possible. Avoiding adverse effects of interventions in education and other fields is especially important in low-income countries, again because there are fewer resources to waste.

### This study

In this study, we created a framework of potential harms of interventions to improve critical thinking about health choices. Our primary objective was to identify potential adverse effects of the Informed Health Choices secondary school intervention, and—as suggested by Weiss ^28^ and Bonell *et al*. ^30^—articulate potential mechanisms. The immediate purpose of the framework was to inform the design of the intervention, to minimize adverse effects, ^66^ the development of outcome measures for assessing such effects; the development of observation and interview guides for exploring potential adverse effects, including potential mechanisms; and the analysis of qualitative data about potential adverse effects. This corresponds with how we and others have used the IHC Key Concepts framework to inform the development of interventions ^65, 66, 69– 74^ and outcome measures, ^75– 77^ to help achieve and evaluate intended effects.

## Methods

### Overview

We developed the framework iteratively, using mixed methods. The process included three main steps: 1) developing an initial framework and criteria for a sensible framework; 2) surveying experts and revising the framework and criteria based on their responses; and 3) interviewing teachers to identify any potential adverse effects still missing from the framework.

### Reflexivity

As part of the greater qualitative evaluation of the IHC secondary school intervention ( Box 1), we have formally applied reflexivity at the individual level through taking reflexive notes, and team-level through structured, reflexive discussions. We describe our subjectivities and report the results of the reflexive strategies, including a team-reflexive statement, in the reports of the separate process evaluations in each country (submitted), and a study across trial settings exploring potential adverse effects. ^78^ The reports of the separate process evaluations also include reflexivity checklists. In the protocol for the cross-trial qualitative study, we incorporated reflexive notes in the methods section. ^78^

### Ethics

Participation in this study was voluntary and did not involve likely or serious risks to participants.

The Norwegian Institute of health is the project’s lead partner. As required by the institute—to comply with the European General Data Protection Regulation—we have completed a data privacy impact assessment each for the entire development and evaluation stages of the project, including this study. The Data Protection and Chief Information Security Officers at the institute provided feedback on the assessments. Furthermore, as required by the Research Council of Norway, we have created a data management plan for the entire project, which we are updating continuously and will submit to the council at the end of the project. Regional Committee for Medical Research Ethics, in Norway, waived the need for ethical approval as the project was not considered health research (reference number 30713, Oct. 10, 2019).

In Kenya, we obtained ethics approval from Masinde Muliro University of Science and Technology Institutional Ethics Review Committee (MMUST/IERC/75/19, Sept. 20, 2019; MMUST/IERC/146/2020, Jan. 5, 2021; MMUST/IERC/018/2022, March 23, 2022), and from the Kenya National Commission of Science and Technology Institute (license number NACOSTI/P/19/1986, Oct. 24, 2019; NACOSTI/P/21/8103, Jan. 6, 2021; NACOSTI/P/22/18813, July 18, 2022), as well as approval from the Ministry of Education, nationally and at the county-level (CDE/KSM/GA/3/24 IV/78, Dec. 23, 2019; CDE/KSM/GA/3/24 V IV/84, Jan. 8, 2020), and from the Teachers Service Commission (KSM/TSC.CD/W/SHOP/VOL.3/146, Jan. 9, 2020). In Rwanda, we obtained ethics approval from the Rwandan National Ethics Committee (approval number 916/RNEC/2019, Nov. 21, 2019; 1019/RNEC/2020, Dec. 22, 2020; 41/RNEC/2022, Feb. 25, 2022; 236/RNEC/2022, Aug. 22, 2022). In Uganda, we obtained ethics approval from the School of Medicine research ethics committee at the Makerere University College of Health Sciences (REC REF 2020-139, Aug. 12, 2020; Aug. 23, 2021), and from the Uganda National Council for Science and Technology (HS916ES, Sept. 23, 2020).

Survey respondents consented to participation by volunteering to submit their responses. When first submitting the manuscript for publication, we also sent it to the survey respondents. We asked them to contact us if they had any concerns. We have not published data that could be used to individually identify them, and we have removed the names of colleagues mentioned in their responses. We obtained written consent from the interview participants for the entire development stage of the project, including being recorded.

### Development of initial framework and criteria

We first developed an initial framework of potential adverse effects of the IHC secondary school intervention, including descriptions of their potential mechanisms and corresponding beneficial effects (potential beneficial effects in place of potential adverse effects, not necessarily the converse of the adverse effect), as well as criteria for a sensible framework. ^79^

We described the potential mechanisms to help identify potential adverse effects, a method suggested by Bonell *et al*. ^30^ and Weiss, ^28^ but also to help identify the corresponding beneficial effects, and inform the development of the intervention towards achieving those corresponding benefits. We based the initial version of the framework on two relevant frameworks: the framework of potential adverse effects of public health interventions developed by Lorenc and Oliver, ^61^ and a framework of potential adverse effects of the IHC primary school intervention, from the process evaluation associated with the trial of the that intervention. ^64^ MO extracted categories of adverse effects, and specific effects. In the initial framework, he included each category or effect if it seemed relevant to the IHC secondary school intervention, and adjusted it if it seemed necessary, or removed it if it seemed irrelevant. AO checked MO’s framework against the other frameworks.

To guide our subjective judgements about what to include in the framework and how, and to make those judgements transparent, we developed criteria for a sensible framework. ^79^ We based the initial criteria for a sensible framework on relevant criteria for sensible health indexes developed by Feinstein. ^80^ In previous work, AO and others have found the latter to be a helpful starting point for developing various tools. ^81, 82^

### Survey of experts, and revision of the initial criteria and framework

We developed a survey for collecting expert feedback on the first version of the framework and criteria, using Nettskjema, an online survey tool developed and hosted by the University of Oslo. ^83^ For a list of all the items and response options, and how the survey appeared to respondents, see the extended data for this study. ^79^

The survey included a series of five-point Likert-scale items assessing the degree to which respondents agreed with eight statements, respectively. ^79^ The statements were based on the initial criteria for a sensible framework, and our initial objectives ^79^:

1. The objectives of the framework are clear and meaningful.
2. The criteria for a sensible framework are clear and meaningful.
3. The framework includes all potential adverse effects that are relevant, logical, conceivable, and important.
4. The framework excludes all potential effects that are not adverse, or are irrelevant, illogical, inconceivable, or unimportant.
5. The structure, organization and presentation of the framework are clear and logical.
6. The framework is as simple as possible without being oversimple.
7. The theoretical mechanisms are clear, logical, and consistent with any relevant evidence.
8. The framework provides a sensible starting point for a generic framework for preventing and evaluating potential adverse effects of critical thinking interventions.

The response options were “Strongly disagree”, “Disagree”, “Neither agree nor disagree”, “Agree”, and “Strongly agree”. If a participant selected one of the first four options, a mandatory, open-ended item appeared, prompting them to explain why they did not strongly agree. At the end of the survey, there were three optional, open-ended items for providing references for “building comparative understanding across similar interventions”, as per the strategy suggested by Bonell *et al*. ^30^; references to other relevant literature; or additional comments or questions.

MO emailed a request for feedback to external experts, as well as the research team. He attached the initial framework and criteria and included a link to the survey. ^79^

The external experts included health and education researchers; other non-researcher educationalists, including teachers and curriculum developers; and information researchers and designers. Recipients were based in low-, middle-, and high-income countries. We did not have a target number of respondents. We contacted all 51 members of our international advisory network, and all 19 other external experts recommended by respondents. In a protocol for stakeholder engagement, we describe our approach to establishing the international advisory network (“panel”). ^84^ MO emailed members of the team and advisory network once as a group and followed up individually with those who did not respond to the initial request. He emailed the 19 other external experts as individuals or individual research groups, at least once.

We analyzed the quantitative survey data using descriptive statistics. MO conducted a thematic analysis of the qualitative survey data. ^85^ The analysis included six steps:

1. organizing the data by questionnaire item, in a spreadsheet,
2. coding each data point with an initial theme emerging from the data,
3. reviewing and revising the codes and themes,
4. organizing the data by theme, in a document,
5. for each theme, suggesting what changes to make to the criteria or framework, if any, and
6. revising the analysis based on feedback from co-investigators.

MO then made the agreed-upon revisions to the criteria and framework.

### Interviews with teachers

We conducted individual interviews with teachers to identify any potential adverse effects missing from the revised framework. We recruited an initial convenience sample of one teacher each from Rwanda, Kenya, and Uganda, the countries in which we developed and evaluated the intervention ( Box 1). We recruited teachers that were proficient in English, and either able to join an online interview on their own, or based close enough to the local research team that a member of the team could help them join. After the third interview, we decided whether additional interviews would be helpful for this study.

We included teachers familiar with the project through participation in an IHC teacher network in their country ^84^ and piloting prototypes of the digital resources. ^66^ We excluded teachers unfamiliar with the project since they were unlikely to have experience teaching critical thinking based on findings from the context analyses ( Box 1). ^18– 20^

We developed an interview guide that we revised after each interview. For the final version of the guide, see the extended data. ^79^ We expected the interview topic—potential adverse effects (“disadvantages”) of teaching critical thinking—would be unfamiliar or strange to teachers. Moreover, we expected that they might find the topic uncomfortable, since they might experience it as being asked to criticize themselves, their colleagues, education authorities, or us, the research team. Therefore, we first asked about potential beneficial effects (“advantages”) of teaching critical thinking. Furthermore, we opted for individual interviews, rather than group interviews, since individual interviews are logically more appropriate for exploring knowledge that is taken for granted and not readily articulated and exploring sensitive topics. ^86^

MO led the interviews via video chat, using Zoom. In two of the three interviews, at least one of the IHC team members in the relevant country joined in-person (RS in Uganda) or via video chat (FC and BN in Kenya), to observe and take additional notes. We video-recorded all the interviews.

MO transcribed participants’ responses to questions about potential adverse effects of teaching critical thinking, then deleted the recordings. He entered those extracts, as well as all notes, into a spreadsheet. He linked the extracts and notes to relevant outcomes in the framework. MO summarized the teachers’ comments about each outcome.

## Results

The results of this study include the following: findings from the survey of experts; the revised criteria for a sensible framework; the revised framework, including an overview (a series of six tables) and descriptions of the potential mechanisms; and findings from the interviews with teachers.

### Survey responses

We received 42 survey responses from people with expertise in:

• Developing and evaluating educational interventions, in general and specifically within critical thinking
• Developing and evaluating health interventions, including public health interventions
• Teaching critical thinking, in general and specifically about health

Four of the survey respondents (10%) were members of our team. In other words, just over half of the external recipients responded to the survey (38/70, 54%). All but one of those respondents were members of our advisory network. A few of the experts we contacted chose to give qualitative feedback via email, in addition to the survey (3 respondents) or instead (4 respondents). The four who provided feedback via email instead of the survey were external and not members of the advisory network. Five of the survey respondents also provided feedback in copies of the circulated document (Supplementary file 1).

Most of the survey respondents were researchers (33 of 42, 79%). Many of those respondents were also educators or health professionals. In total, twenty-four (57%) of the respondents worked primarily in public health, health literacy, or evidence-based health care, and 15 (36%) in education. The remaining three respondents worked primarily as information researchers and designers. Thirty-one (74%) of the survey respondents were based in high-income countries. Twenty-four (57%) were based in Europe, 8 (19%) in Africa, 5 (12%) in North America, and 5 (12%) in Australia, Central or South America, or Asia.

For the full results for the Likert-scale items, see the underlying data for this study. ^87^ Those results are summarized in Box 2. They suggest respondents overall approved of the initial framework and criteria.

Box 2. **Quantitative survey results.**

• 41 respondents (98%) agreed or strongly agreed that the objectives of the initial framework were clear and meaningful.
• 40 (95%) agreed or strongly agreed that the initial criteria for a sensible framework were clear and meaningful.
• 36 (86%) agreed or strongly agreed that the initial framework included all potential adverse effects relevant, logical, conceivable, and important.
• 36 (86%) agreed or strongly agreed that it excluded all potential effects that are not adverse, or are irrelevant, illogical, inconceivable, or unimportant.
• 40 (95%) agreed or strongly agreed that the structure, organization and presentation of the framework were clear and logical.
• 41 (98%) agreed or strongly agreed that the framework was as simple as possible without being oversimple.
• 39 (93%) agreed or strongly agreed that the initial descriptions of the theoretical were clear, logical, and consistent with any relevant evidence.
• All respondents agreed or strongly agreed that the initial framework provided a sensible starting point for a generic framework for preventing and evaluating potential adverse effects of critical thinking interventions.

However, the qualitative survey data ^87^ suggested there were important problems, and led to substantial revisions of both the criteria and framework. For all those data, and an overview of the seven themes and 36 sub-themes emerging from the data, see the underlying data. ^87^ Therein, the data are organized alphabetically by theme and sub-theme, together with our responses, including the specific revisions we made. ^87^ We have also included a list of additional changes that we made to the initial framework. ^87^ The survey responses included few references to relevant literature. ^87^

### Revised criteria for a sensible framework

Box 3 shows the revised criteria for a sensible framework. For the qualitative survey feedback on the initial criteria and changes we made to the criteria, see the underlying data. ^87^

Box 3. **Revised criteria for a sensible framework.**

Each category of potential adverse effects is clear and logical.

Each potential adverse effect is clear, logical, and likely to be important to participants or other stakeholders.

Each corresponding beneficial effect is clear and logical.

The amount of content in the framework is manageable.

The organization of the framework is clear and logical.

The presentation of the framework is clear and logical.

### Revised framework

The revised framework has two parts. The first part is an overview: a series of six tables presenting the categories of adverse outcomes with definitions ( Table 1), outcomes within those categories ( Table 2), definitions of those outcomes ( Table 3), sub-outcomes ( Table 4), potentially affected individuals, groups, and populations ( Table 5), and corresponding beneficial outcomes ( Table 6). The second part of the framework is descriptions of potential mechanisms for the effects, with examples. The categories of adverse effects ( Table 1) correspond with the categories in the framework developed by Lorenc and Oliver, ^61^ with three modifications. First, we changed “Direct harms [to physical health]” to “Decision-making harms”. We did not identify any potential harms of the IHC secondary school intervention directly to physical health. However, it is possible that they intervention could lead to decisions that in turn lead to physical harm. Second, we changed “Opportunity cost harms” to “Waste”, which is more succinct. Third, we added the category “Other harms”, since there might be categories that we have failed to consider. We included all the adverse effects in the framework of potential adverse effect of the IHC primary school intervention, ^64^ with one modification: we replaced both “Nihilism or cynicism” and “Shortened enjoyment of the innocence of childhood” with “Pessimism”. We did this because both “nihilism” and “cynicism” have varied definitions; the relevant definitions are captured by “pessimism”; and shortened enjoyment of the innocence of childhood would be a consequence of pessimism (Supplementary file 5).

**Table 1.** Categories of adverse outcomes.

Category	Definition
*Decision-making harms*	Behaviors and beliefs that might contribute to poor choices
*Psychological harms*	Uncomfortable thoughts and feelings
*Equity harms*	Inequities in the distribution or size of effects
*Group and social harms*	Harmful interactions between individuals, groups, populations, and systems
*Waste*	Waste of time and resources
*Other harms*	Other adverse outcomes than those in the categories above

**Table 2.** Adverse outcomes by category.

Category	Adverse outcome
*Decision-making harms*	Misunderstanding
	Misapplication of learning
*Psychological harms*	Distrust ^1^
	Pessimism ^2^
	Cognitive dissonance
	Work/Schoolwork-related stress
*Equity harms*	Benefit-based inequity
	Harm-based inequity
*Group and social harms*	Conflict
*Waste*	Wasted time or resources
*Any category*	Other harms than those above

^1^ Distrust might also be a decision-making harm, or group or social harm.

^2^ Pessimism might also be a decision-making harm.

**Table 3.** Definitions of adverse outcomes.

Adverse outcome	Definition
*Misunderstanding*	Incorrect understanding of a concept or example explained in the intervention
*Misapplication of learning*	Incorrect or unnecessary application of a skill or knowledge learned from the intervention
*Distrust*	Feeling that a person or organization cannot be relied upon
*Pessimism*	Inclination to believe that the application of skills or knowledge learned from the intervention is impossible or useless
*Cognitive dissonance*	Experience of inconsistent beliefs
*Work/Schoolwork-related stress*	Mental or emotional strain from work or schoolwork
*Benefit-based inequity*	Inequity due to the distribution or size of a beneficial effect of the intervention
*Harm-based inequity*	Inequity due to the distribution or size of an adverse effect of the intervention
*Conflict*	Unconstructive argument between two or more parties
*Wasted time or resources*	Use of time or resources on the intervention that would be better spent on other activity
*Other harms*	Other adverse outcomes than those specified above

**Table 4.** Sub-outcomes.

Adverse outcome	Sub-outcomes	
*Distrust*	towards	• Health professional • Health researcher • Family • Teacher • Other
*Conflict*	involving	• Participant ^1^ • School • Family • Social networks ^2^ • Health services ^3^ • Researcher • Other
*Benefit-based inequity*	between	• High and low-achieving students • High and low-resourced students • High and low-resourced schools • Other

^1^ Student or teacher.

^2^ Friends, peers, colleagues, religious leaders, and others.

^3^ Health professionals and others.

**Table 5.** Affected individuals, groups, and populations, by adverse outcome.

Adverse outcome	Directly affected	Indirectly affected
*Misunderstanding*	Participant ^1^	Family, social networks ^2^, and health services ^3^
*Misapplication of learning*	Participant ^1^	Family, social networks ^2^, and health services ^3^
*Distrust*	Participant ^1^	Family, social networks ^2^, and health services ^3^
*Pessimism*	Participant ^1^	Family, social networks ^2^, and health services ^3^
*Cognitive dissonance*	Participant ^1^	Family and social networks ^2^
*Work/Schoolwork-related stress*	Participant ^1^	Family and social networks ^2^
*Benefit-based inequity*	Participants ^1^ or schools	Families and social networks ^2^
*Harm-based inequity*	Participants ^1^ or schools	Families and social networks ^2^
*Conflict*	Participant ^1^/school and second party	Family, social networks ^2^, health services ^3^, and researchers
*Wasted time or resources*	Participants ^1^ or schools	Families and social networks ^2^
*Other*	Any	Any

^1^ Student or teacher.

^2^ Friends, peers, colleagues, religious leaders, and others.

^3^ Health professionals and others.

**Table 6.** Corresponding beneficial outcomes.

Adverse outcome	Corresponding beneficial outcome ^1^
*Misunderstanding*	Understanding
*Misapplication of learning*	Correct and necessary application of learning
*Distrust*	Healthy skepticism
*Pessimism*	Optimism
*Cognitive dissonance*	Cognitive coherence
*Work/Schoolwork-related stress*	None
*Benefit-based inequity*	Benefit-based equity
*Harm-based inequity*	None
*Conflict*	Constructive discussion
*Wasted time or resources*	Worthwhile use of time and resources

^1^ The corresponding beneficial effect (an increase in the corresponding beneficial outcome) is a potential beneficial effect in place of the potential adverse effect, not necessarily the converse of the adverse effect.

*Misunderstanding*

Educational interventions might cause misunderstandings of concepts or examples, which participants might then apply or “transfer” ^88^ to their daily lives. Such effects are possible if the concepts or examples are unclear to participants due to a problem with how they are explained or used. With the IHC secondary school intervention, examples of health claims might be misunderstood as advice if it is unclear how the intervention is different from typical public health interventions, which encourage certain choices, such as getting screened for a disease, ^89^ as opposed to helping people learn general decision-making skills ( Box 4).

Box 4. **Examples of potential misunderstandings caused by the intervention.**

**Misunderstanding a concept**

In terms of the IHC Key concepts about the reliability of claims and research evidence, a participant might misunderstand “unreliable” as meaning the same as “false” or “incorrect”. This is possible if the meaning of “unreliable” is unclear due to a problem with its explanation or use in the training or resources. In their daily life, when faced with an unreliable claim about the effects of a health intervention, the participant might then assume the claim is simply false and the intervention does not have the claimed effects. In fact, the intervention might have the claimed effects, despite the claim being unreliable. Moreover, there might be an alternate, reliable basis for the claim. For example, when faced with a claim about the helpful effects of a vaccine based on anecdotal evidence, the participant might assume the claim is false and the vaccine is wasteful, if not harmful. However, the vaccine might still be helpful, and there might be reliable evidence showing it is helpful.

**Misunderstanding an example**

It is possible that participants will interpret examples of reliable claims as advice. This is possible if the explanation or use of an example is unclear. For example, a participant might interpret an example about evidence showing helpful effects of painkillers as advice that they should always use painkillers when in pain. Vice versa, they might interpret an example about evidence showing side effects of painkillers as advice that they should never use painkillers.

*Misapplication of learning*

An educational intervention might improve skills in the learning context (i.e., the training or lessons), but cause misapplication of those skills in other contexts, most importantly participants’ daily lives. Such “mis-transfer” of learning—as opposed to transfer of mislearning (see “Misunderstanding”)—is possible if limitations of the intervention are not addressed clearly, in the intervention. The IHC secondary school intervention focuses on a limited number of IHC Key Concepts ( Box 1) and does not address other relevant and important skill sets, such as the ability to search for relevant and reliable information.

For example, a participant might understand the concept that randomization is the only way to control for unknown confounding. However, the intervention does not focus on concepts about blinding. If this limitation of the intervention is unclear, when in daily life the participant is faced with evidence from a randomized, but unblind trial, with a high risk of bias, they might then assume the evidence is more reliable than it is.

Misapplication of learning here includes “overtransfer” ^88^: technically correct, but unnecessary application of learning. For example, a participant might spend time and energy assessing the basis for a health claim that could simply be ignored because the intervention in the claim is unavailable to them.

*Distrust*

Interventions to improve critical thinking about health choices might cause participants to become distrustful of certain groups or individuals. Such effects of the IHC secondary school intervention are possible if the difference between the basis for a claim and its source is unclear to participants, as well as why the basis, not the source, determines the reliability of the claim.

The IHC secondary school intervention might cause distrust towards health professionals or researchers. This is because when explaining ways in which research can be unreliable, such as random error, we disproportionately use examples of unreliable research evaluating the effects of “Western”, “modern”, “conventional”, or “academic” medicine, as opposed to “traditional”, “herbal” or “natural” medicine. These examples typically suggest the intervention is helpful, when really it is or might be ineffective or harmful.

There are two reasons for this disproportion. First, participants might be deeply invested in beliefs about “traditional” and “herbal” care. If so, they might become defensive if we use an example suggesting such beliefs are unreliable, and their defensiveness might prevent learning. Moreover, they might assume the intervention is on the side of “Western” medicine and the pharmaceutical industry of which they might already be distrustful for understandable reasons, starting with colonialism. ^90^

Second, to effectively explain concepts, our findings and experiences from the development of the IHC primary and secondary school interventions suggest it is important to use real, familiar, and relevant examples, showing that the concepts are important to participants’ daily lives. However, there often is a lack of research evaluating the effects of traditional and herbal interventions that are common in the participants’ contexts.

Increased distrust might lead to poor decisions, by causing participants to dismiss reliable and relevant advice or evidence, making it a potential decision-making harm as well as a potential psychological harm. Moreover, it might lead to conflict, making it a potential group or social harm.

In some cases, distrust might be a decision-making benefit. However, any source might provide reliable or unreliable information about the effects of a health intervention. Furthermore, aiming to increase trust or distrust in particular sources might backfire. For example, aiming to increase trust in health authorities or decrease trust in practitioners of “traditional” medicine could make the IHC secondary school intervention seem like propaganda for “Western” medicine.

Therefore, the IHC secondary school intervention is not intended to increase or decrease trust in information about types of interventions, nor information from certain sources. Rather, it is intended to increase healthy skepticism towards all claims and research evidence. If the intervention is effective, students should be more likely to seek advice from health authorities, assuming those authorities provide reliable information. And they should be less likely to seek information from sources that more often provide unreliable information.

*Pessimism*

Interventions to improve critical thinking about health choices might cause participants to feel pessimistic. Such effects of the IHC secondary school intervention are possible if it is unclear to participants how the intervention can help them make better choices in many cases, even though there are relevant and important problems that the intervention cannot solve. Those problems include:

• Many people lack access to effective and safe care. ^14^
• Many common health interventions are harmful or wasteful or have highly uncertain effects. ^14^
• Many health claims are unreliable. ^1^
• Many people are limited in their ability to think critically about health information and choices, including health professionals. ^5– 8, 16, 17^
• Many health research studies are unreliable or wasteful. ^2, 91– 93^
• Citizens have limited influence over decisions about health policies that affect them. ^94^
• Free sources of reliable health information in plain language are limited in number and quality. ^95^
• In school, participants have limited opportunities to learn about how to think critically about health. ^18– 20^

We address some of these problems explicitly in the intervention, and participants might themselves recognize others. They might believe that because of these problems, applying the skills they have learned from the intervention is impossible or useless. This might be more likely in students than teachers, given that students have less experience making independent choices.

Increased pessimism might lead to poor decisions, by causing participants to mistakenly assume an informed choice is impossible or useless, making it a potential decision-making harm as well.

*Cognitive dissonance*

Interventions like the IHC secondary school intervention might lead to uncomfortable cognitive dissonance. This effect is possible if the intervention causes participants to have new beliefs that are inconsistent with prior, deep-rooted beliefs, such as beliefs about the effects of “traditional” medicine (see “Distrust”). The IHC Secondary school intervention might help participants apply a concept to some beliefs about the effects of health interventions, but not others, depending on the participant’s level of emotional, social, or cultural investment in the belief. Recognizing this inconsistency might be uncomfortable.

*Work/Schoolwork-related stress*

Educational interventions might cause work-related stress in participating teachers, and schoolwork-related stress in participating students. Such effects are possible if the intervention is ineffective, inefficient, or inessential, or experienced as such, or if it is too demanding. As mentioned in the introduction: in the process evaluation for the trial of the IHC primary school intervention, some teachers reported stress from the added workload, and teaching something new. ^64^

It might be time-consuming for participants to familiarize themselves with the content of the IHC secondary school intervention—including terminology, concepts, and teaching and learning strategies—as well as the design and functionality of the digital resources. Meanwhile, there is pressure on students, teachers, and schools to prepare as much as possible for official exams, which do not test the ability to apply IHC Key Concepts. ^18– 20^

Furthermore, the IHC secondary school intervention is intended to increase students’ questioning of claims, evidence, and choices. If students more often question teachers’ claims or choices, this too might lead to teachers feeling stressed.

*Inequity*

Educational interventions such as the IHC secondary school intervention might cause or increase inequity. Such effects are possible if there is an unequal size or distribution of beneficial or harmful effects across subgroups. In particular, the intervention might increase inequity amongst students depending on their baseline academic ability, or their socioeconomic background and resources. For example, with terminology used in the intervention might be suitable for some students, but too advanced for others, or some students might receive academic support at home that others do not.

Similarly, the intervention might increase inequity amongst schools depending on available resources. For example, the intervention might have a larger beneficial effect on learning in participants at highly resourced schools. Or it might cause more work-related stress in teachers at low-resource schools who have relatively little training or support.

*Conflict*

Interventions to improve critical thinking about health choices might cause conflicts between different individuals and groups. Such effects are possible if the intervention causes participants to question other people’s claims, beliefs, or choices, and those people become irritated or defensive. As mentioned in the introduction, this was a concern expressed by teachers and parents in relation to the IHC primary school intervention. ^64^

Interventions like the IHC secondary school intervention might cause conflict between students and authority figures in particular, such as teachers or parents. For example, a parent might be deeply invested in beliefs about the effects of “traditional” or “herbal” care (see “Distrust”). If the child questions such beliefs—especially in a way that is experienced as disrespectful—this might lead to conflict.

*Wasted time or resources*

With any intervention, there is an opportunity cost. Such effects are possible if the intervention is ineffective or inessential, or experienced as such. As mentioned under “Work/Schoolwork-related stress”, there is pressure on students, teachers, and schools to prepare as much as possible for official exams. ^18– 20^ This is important in terms of waste since any time spent on an ineffective or inessential intervention could have been spent on preparation for exams.

*Other potential adverse outcomes*

The IHC secondary school intervention and other interventions to improve critical thinking about health choices might have adverse effects that are not specified in the framework. This includes effects that we have considered and chosen not to specify, because they seem illogical, such as an increase in bullying, ^87^ as well as any effects that we have failed to consider.

### Interview findings

We interviewed one teacher in each of the three countries where we evaluated the IHC secondary school intervention ( Table 7). After the third interview, we decided additional interviews would not be helpful for this study, because of limited variation in the feedback across interviews. The interview data are included in the underlying data. ^87^ Teachers’ comments about potential effects are summarized in the extended data. ^79^ The teachers we interviewed did not identify any potential adverse effects not already explicit in the revised framework.

**Table 7.** Interview participants.

	Teacher 1	Teacher 2	Teacher 3
*Country*	Uganda	Kenya	Rwanda
*Years of teaching experience*	10	4	10
*Subjects*	Biology, chemistry	Biology, agriculture	Physics
*Forms (grades or years)*	1-4 (entire lower secondary)	1-4 (entire lower secondary)	4-6 (entire upper secondary)
*School location*	Urban	Semi-urban	Urban
*School type*	Private	Public	Semi-public (“government-aided”)

We organized the interview data in five main themes ( Table 8). Within the theme about potential adverse effects (“Disadvantages”), we organized the data into 10 sub-themes ( Table 8). Each sub-theme is a potential adverse outcome, or a factor in the potential mechanism of an adverse effect.

**Table 8.** Themes and sub-themes from analysis of interview findings.

Theme	Sub-theme
*Advantages [of teaching critical thinking]*	[No sub-themes]
*Development of [IHC secondary school] intervention*	[No sub-themes]
*Disadvantages [of critical thinking]*	Cognitive dissonance Conflict Distraction Distrust Exams Lack of effect Misapplication [of learning] Overconfidence Pessimism Stress
*Interview [experience]*	[No sub-themes]
*Other initiatives [to teach critical thinking]*	[No sub-themes]

Teacher 1 reported two “worries” or “challenges” for teaching critical thinking, which we linked to the outcomes “Wasted time or resources” and “Work-related stress”. Teacher 2 reported experiencing stress and wasted time in the pilot, and suggested conflict was another potential adverse effect, although he had not experienced or observed it. Teacher 3 initially said there were no potential adverse effects of teaching critical thinking: *“When you think critically, you can go far, but when you don’t think critically, I think you can go nowhere. So, [there is] no disadvantage.*" When we asked him about the potential adverse effects suggested by Teachers 1 and 2, he suggested those effects were either not possible, or acceptable and easily addressed.

## Discussion

### Strengths of the study and framework

Strengths of this study include the iterative approach, the use of mixed methods, interdisciplinary collaboration, and stakeholder feedback. Strengths of the framework include its comprehensiveness, and that it is open-ended and “living”.

Interdisciplinary collaboration is important when trying to help people learn how to think critically. ^25, 96, 97^ We took advantage of an interdisciplinary advisory network systematically established in a separate study. ^84^ The survey respondents, including members of the network and others, had expertise from a wide variety of fields and country contexts.

We took a more rigorous approach to developing the framework than the approach taken by Lorenc and Oliver, ^61^ or the one taken in the process evaluation for the trial of the IHC primary school intervention. ^64^ Stratil *et al*. took a rigorous approach to developing the CONSEQUENT framework, which was reported after the completion of this study. ^62^ However, the CONSEQUENT framework is broader, and does not address potential adverse effects of educational interventions specifically. The framework developed in this study is more comprehensive in terms of educational interventions than both the two on which it is based ^61, 64^ and the CONSEQUENT framework. ^62^

### Limitations of the study and framework

A limitation of this study is the limited response rate to the survey, and the limited sample of interviewees. Important limitations of the framework include trade-offs favoring manageability over comprehensiveness, and the use of terminology with different meanings or interpretations depending on the context. Furthermore, there were few evaluations of potential adverse effects of educational interventions on which to base the framework, and the generic value of the framework is uncertain.

In the evaluation of the IHC secondary school intervention, we are addressing the limitations of the framework by using complementary mixed methods; conducting interviews that are semi-structured and including an inductive component in our analysis; and being transparent and showing appropriate caution in the interpretation of results.

### Implications

The framework developed in this study shows that interventions to improve critical thinking about health choices might have a variety of important adverse effects. We have used the framework to evaluate potential adverse effects of the IHC secondary school intervention in the process evaluations, ^78, 98– 100^ and in the one-year follow-up assessments for the randomized trials. ^101^ First, based on the interview findings, we preliminarily prioritized three outcomes, for both the quantitative and qualitative evaluations: work-related stress; wasted time or resources; and conflict, especially between students and family. Second, we used the framework to develop observation and interview guide items for the process evaluations, ^98– 100^ and are using it to analyze qualitative data. ^78^ Third, we used it to formulate questions administered together with the measures of intended outcomes, in the one-year follow-up. ^101^

The framework is a “living” tool, which can be improved upon, as well as adapted for the development and evaluation of interventions to improve critical thinking in other fields. ^25^ The framework might also be useful to policymakers, intervention designers, and educators, in addition to researchers.

## Conclusions

In this mixed-methods study, we developed a framework of potential adverse effects of an intervention intended to improve critical thinking about health choices, including potential mechanisms. We are unaware of any similar tool for education research. Overall, survey responses from researchers and other experts were positive to an initial version of the framework. However, responses also included critical feedback that led to substantial revisions. Teachers interviewed about potential adverse effects of teaching critical thinking did not report or suggest any potential adverse effects that were missing from the framework.

We have used the framework in both the qualitative and quantitative evaluations of the IHC secondary school intervention. The framework can be improved upon and adapted. The framework might be of use to other researchers, as well as policymakers, intervention designers, and educators.

Educational interventions warrant rigorous development and evaluation, especially prior to large-scale implementation, not only because the intervention might be ineffective and wasteful, or have paradoxical effects, but because it might cause other adverse effects. Rigorous evaluations of potential adverse effects of educational interventions, as well as efforts to prevent those effects, can be costly. However, these evaluations might come at a small cost compared to the cost of implementing harmful interventions.

## Ethics and consent

Participation in this study was voluntary and did not involve likely or serious risks to participants.

The Norwegian Institute of health is the project’s lead partner. As required by the institute—to comply with the European General Data Protection Regulation—we have completed a data privacy impact assessment each for the entire development and evaluation stages of the project, including this study. The Data Protection and Chief Information Security Officers at the institute provided feedback on the assessments. Furthermore, as required by the Research Council of Norway, we have created a data management plan for the entire project, which we are updating continuously and will submit to the council at the end of the project. Regional Committee for Medical Research Ethics, in Norway, waived the need for ethical approval as the project was not considered health research (reference number 30713, Oct. 10, 2019).

In Kenya, we obtained ethics approval from Masinde Muliro University of Science and Technology Institutional Ethics Review Committee (MMUST/IERC/75/19, Sept. 20, 2019; MMUST/IERC/146/2020, Jan. 5, 2021; MMUST/IERC/018/2022, March 23, 2022), and from the Kenya National Commission of Science and Technology Institute (license number NACOSTI/P/19/1986, Oct. 24, 2019; NACOSTI/P/21/8103, Jan. 6, 2021; NACOSTI/P/22/18813, July 18, 2022), as well as approval from the Ministry of Education, nationally and at the county-level (CDE/KSM/GA/3/24 IV/78, Dec. 23, 2019; CDE/KSM/GA/3/24 V IV/84, Jan. 8, 2020), and from the Teachers Service Commission (KSM/TSC.CD/W/SHOP/VOL.3/146, Jan. 9, 2020). In Rwanda, we obtained ethics approval from the Rwandan National Ethics Committee (approval number 916/RNEC/2019, Nov. 21, 2019; 1019/RNEC/2020, Dec. 22, 2020; 41/RNEC/2022, Feb. 25, 2022; 236/RNEC/2022, Aug. 22, 2022). In Uganda, we obtained ethics approval from the School of Medicine research ethics committee at the Makerere University College of Health Sciences (REC REF 2020-139, Aug. 12, 2020; Aug. 23, 2021), and from the Uganda National Council for Science and Technology (HS916ES, Sept. 23, 2020).

Survey respondents consented to participation by volunteering to submit their responses. When first submitting the manuscript for publication, we also sent it to the survey respondents. We asked them to contact us if they had any concerns. We have not published data that could be used to individually identify them, and we have removed the names of colleagues mentioned in their responses. We obtained written consent from the interview participants for the entire development stage of the project, including being recorded.

## Data Availability

Zenodo. Underlying data for “Development of a framework of potential adverse effects of interventions to improve critical thinking about health choices: A mixed methods study.” https://doi.org/10.5281/zenodo.13934351. ^87^ This project contains the following underlying data:
• Data file 1 Quantitative survey results.pdf • Data file 2 Qualitative survey results.docx • Data file 3 Interview data.xlsx Data are available under the terms of the Creative Commons Attribution 4.0 International license (CC-BY 4.0). Zenodo. Extended data for “Development of a framework of potential adverse effects of interventions to improve critical thinking about health choices: A mixed methods study.” https://doi.org/10.5281/zenodo.13934160. ^79^ This project contains the following extended data:
• Supplementary file 1 Initial framework and criteria.docx • Supplementary file 2 Survey.pdf • Supplementary file 3 Interview guide.docx • Supplementary file 4 Interview findings.docx Data are available under the terms of the Creative Commons Attribution 4.0 International license (CC-BY 4.0).
