# Supplementary material for: Development of a framework of potential adverse effects of interventions to improve critical thinking about health choices: A mixed methods study
Source: F1000Res. 2025 Dec 31;13:1303. Originally published 2024 Oct 31. [Version 2] doi: 10.12688/f1000research.158042.2 (PMC12676117; doi:10.12688/f1000research.158042.2)
Supplement: Peer review history

# Reviewer report: Reviewer response for version 1

**DOI:** 10.5256/f1000research.173580.r340296

**Published:** 2025-12-03

**Authors:** Shelly Melissa Pranic

**Affiliations:** 1 Cochrane Croatia, Split, Croatia

In countries that were former colonies, it is understandable that initiatives from the former ruling countries would be viewed with distrust. As adverse effects due to interventions are possible, distrust will be among them in settings where authorities' and researchers' intentions were unscrupulous in the past. Involving community members in the design and conduct of trials to test the effectiveness of traditional versus “Western” or “academic” treatments could be a way to decrease mistrust. The authors provide a framework to evaluate the potential adverse effects of the IHC school interventions, which is important for any public health intervention.

 I wonder about the involvement of high school students in the development of the framework. Although the authors state that students do not have sufficient experience in recognizing that interventions could be useless or impossible, why weren’t students and parents involved in the development of the “living” framework? Parents’ influence on the children’s decisions after exposure to the IHC intervention should also be considered by incorporating parental views. Diverse stakeholders, not just academic experts, would add more lived experience expertise to the framework.

 Could the authors state whether there is a possibility and plans to include those (especially children’s) voices (in the form of interviews) in the living framework that weren’t a part of the initial framework development?

Is the work clearly and accurately presented and does it cite the current literature?

Yes

If applicable, is the statistical analysis and its interpretation appropriate?

Not applicable

Are all the source data underlying the results available to ensure full reproducibility?

Yes

Is the study design appropriate and is the work technically sound?

Yes

Are the conclusions drawn adequately supported by the results?

Yes

Are sufficient details of methods and analysis provided to allow replication by others?

Yes

Reviewer Expertise:

Health services research, public health, epidemiology, meta-research

I confirm that I have read this submission and believe that I have an appropriate level of expertise to confirm that it is of an acceptable scientific standard.

# Reviewer report: Reviewer response for version 1

**DOI:** 10.5256/f1000research.173580.r343094

**Published:** 2025-06-16

**Authors:** Anke Steckelberg

**Affiliations:** 1 Interdisciplinary Center for Health Sciences, Institute of Health and Nursing Science, Martin-Luther-Universitat Halle-Wittenberg, Halle (Saale), Saxony-Anhalt, Germany

The manuscript addresses a very relevant topic - side effects of educational interventions. The topic has so far been insufficiently addressed in the literature.

 The authors' aim was to develop a framework of potential side effects of IHC interventions including potential mechanisms. The framework  may later on  inform the development of educational interventions. . 

 Please find my comments below:

**General comments:**

 -The term stakeholder should be replaced by interest-holder as recently suggested by Akl et al. 

 (refer to 1. ) 

 - The authors should include a reporting statement., particularly with regard to the qualitative analyses. In particular, there is a lack of information on the authors with regard to previous experience with qualitative research and relationships with the interview partners. This should be reflected in the discussion.

**Methods:**

 - The second step comprised an expert survey. Were all experts familiar with the IHC project? Please clarify.

 - In the  third phase the authors conducted interviews with teachers who were familiar with the IHC project. Were any other inclusion criteria considered beside being familiar with the IHC project? Experiences in teaching critical thinking? (in years); Please clarify.

**Discussion:**

 - The authors did not include parents into the third phase of their study? Some side effects of the intervention may become apparent, particularly in the family environment. 

Is the work clearly and accurately presented and does it cite the current literature?

Yes

If applicable, is the statistical analysis and its interpretation appropriate?

Not applicable

Are all the source data underlying the results available to ensure full reproducibility?

Yes

Is the study design appropriate and is the work technically sound?

Yes

Are the conclusions drawn adequately supported by the results?

Yes

Are sufficient details of methods and analysis provided to allow replication by others?

Partly

Reviewer Expertise:

critical health literacy; evidence-based medicine; evidence-based health information; informed choice

I confirm that I have read this submission and believe that I have an appropriate level of expertise to confirm that it is of an acceptable scientific standard.

## References

[1] : “Interest‐holders”: A new term to replace “stakeholders” in the context of health research and policy. *Cochrane Evidence Synthesis and Methods* .2024;2(11) : 10.1002/cesm.70007 10.1002/cesm.70007 PMC1179595740475280
